# Fabrication of Hybrid Membranes Containing Nylon-11 and Organic Semiconductor Particles with Potential Applications in Molecular Electronics

**DOI:** 10.3390/polym12010009

**Published:** 2019-12-19

**Authors:** María Elena Sánchez-Vergara, Elizabeth Guevara-Martínez, Alejandra Arreola-Castillo, Alejandra Mendoza-Sevilla

**Affiliations:** Department of Engineering, Universidad Anáhuac México, Avenida Universidad Anáhuac 46, Col. Lomas Anáhuac 52786, Huixquilucan, Estado de México, Mexico; elizabeth.guevara@anahuac.mx (E.G.-M.); ale.arreola99@gmail.com (A.A.-C.); amendoza_sevilla@hotmail.com (A.M.-S.)

**Keywords:** molecular semiconductor, semiconductor membrane, high-vacuum evaporation, thin film, optoelectronic properties

## Abstract

Chemical degradation is a major disadvantage in the development of organic semiconductors. This work proposes the manufacture and characterization of organic semiconductor membranes in order to prevent semiconductor properties decreasing. Semiconductor membranes consisting of Nylon-11 and particles of π-conjugated molecular semiconductors were manufactured by high-vacuum evaporation followed by thermal relaxation. Initially, and with the aim of obtaining semiconductor particles, bulk heterojunction (BHJ) was carried out using green chemistry techniques between the zinc phthalocyanine (ZnPc) and the zinc hexadecafluoro-phthalocyanine (F_16_ZnPc) as *n*-type molecular semiconductors with the *p*-type molecular semiconductor dibenzotetrathiafulvalene (DBTTF). Consequently, the π-conjugated semiconductors particles were embedded in a Nylon-11 matrix and characterized, both structurally and considering their optical and electrical properties. Thin films of these materials were manufactured in order to comparatively study the membranes and precursor semiconductor particles. The membranes presented bandgap (E_g_) values that were lower than those obtained in the films, which is an indicator of an improvement in their semiconductor capacity. Finally, the membranes were subjected to accelerated lighting conditions, to determine the stability of the polymer and the operating capacity of the membrane. After fatigue conditions, the electrical behavior of the proposed semiconductor membranes remained practically unaltered; therefore, they could have potential applications in molecular electronics. The chemical stability of membranes, which did not degrade in their polymer compound, nor in the semiconductor, was monitored by IR spectroscopy.

## 1. Introduction

Semiconductors are emerging as systems for biophysical tools and biomedical devices. Devices that integrate semiconductors have a rich set of physical properties that make them desirable targets for the design of next-generation biomedical devices. In addition, their intrinsic small size gives rise to both high spatial resolution and minimal invasiveness [1,2,3,4]. Most used in smart implantable drug delivery systems [5,6], these devices release any biological agent that is introduced into them in a small polymeric compartment called a transporter. Another application is as sensors in noninvasive procedures, mainly because of their size and semiconductor qualities, making them susceptible to chemical and electrical changes [1,7,8]. The selection of an inorganic or organic semiconductor is very important, since the operation of the device depends on the chemical, optical, and electrical characteristics of the semiconductor. The synthesis of organic semiconductors based on metal phthalocyanines (MPcs) is thus very important in the development of biomedical devices, due to their excellent photoelectric response, optical properties, and high chemical and thermal stability. Moreover, they are not toxic or contaminant molecules, which means their manufacture is a suitable form of green chemistry. Green chemistry is a revolutionary way of approaching the synthesis of processes and products that reduces their risks to both human health and the environment. This has given rise to the possibility of generating electronic devices whose manufacture is more ecological. Unfortunately, the performance of electronic devices is compromised under environmental conditions, so protection against degradation is necessary. Polymeric membranes are an exceptional option for isolating MPcs without affecting their charge transport properties [9]. Nylon-12 is known to be a polymeric biomaterial, and is commonly used as a cartilage replacement. However, it is necessary to study other types of polyamides that can be used in the manufacture of biomedical systems [10]. This work focuses on MPcs semiconductors that are deposited on Nylon-11 (PA-11) membranes. Nylon is a major synthetic polyamide, and is mostly used for its net-like structure in membranes, due to the hydrogen bonds in the chain, and its high resistance in the direction of the fiber. Polyamide-11 (PA-11) is unique among petroleum-based nylons and other conventional plastics because it is made from castor oil, a 100% bio-renewable material. It is a high-performance, semi-crystalline, thermoplastic polymer. PA-11 has low CO_2_ emissions and low global warming potential. PA-11 provides superior impact properties to other nylons at ambient temperatures, as well as flexibility, high abrasion resistance, high thermal stability, low specific gravity, excellent chemical resistance, low moisture absorption, and the capability to be processed at a wide range of temperatures. PA-11 also has excellent dimensional stability and maintains its physical properties at a wide range of temperatures, and in a wide range of environments [11,12]. With respect to chemical environments, Thomas Graham was the first to describe the solution-permeation model for polymeric membranes, and his research about porous membranes led to Graham’s Law of Effusion [13]. It was observed that nylon has lower permeability for many gases in comparison to similar materials [14,15,16,17,18]. On the other hand, it has been used for electrospun fibers as support material, resulting in a lack of the mechanical strength that polyamides membranes may possess [19]. These physical and chemical properties make nylon membranes specifically useful as components for electronic devices of biomedical structures. The polymer-membranes isolate semiconductor nanoparticles that are finely dispersed in the polymer. From one perspective, the polymer matrix plays an important role in the optical and electric properties of micro-electronic devices. Conversely, physical attributes such as optic or electric properties may be modified, depending on the particle’s size, shape, and nature [20]. For biomedical applications, inorganic semiconductor nanoparticles are not normally degradable and are potentially toxic. The synthesis of organic semiconductor particle (ONs) membranes has been regarded as a better candidate than its inorganic counterparts, as a result of their structural variability, lower toxicity, cost efficiency, and biodegradable potential [21]. According to the regime where the ONs operate inside the devices, they can be divided into (i) π-conjugated molecular semiconductors (*p*-type), (ii) π-conjugated molecular semiconductors (*n*-type), and (iii) π-conjugated molecular semiconductors ambipolar. This last type of semiconductor is characterized by its formation from a mixture of *n*-type and *p*-type semiconductors [22], and their function within the devices consists of generating balanced mobilities of electrons and hollow.

**Figure 1:**
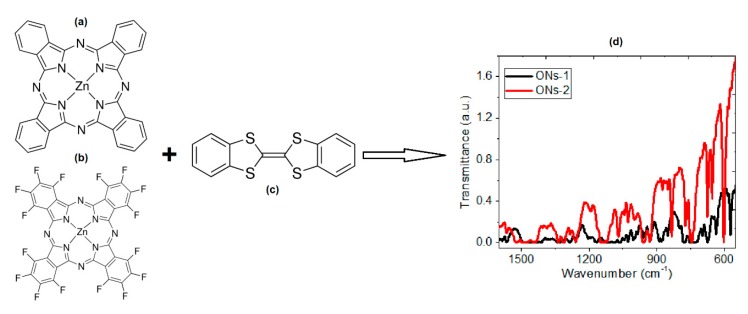
Molecular structure for: (**a**) ZnPc, (**b**) F_16_ZnPc, (**c**) DBTTF and (**d**) IR spectrum for ONs.

The aim of the present work is to manufacture and characterize semiconductor membranes that consist of a Nylon-11 matrix and ONs of zinc phthalocyanines (ZnPc and F_16_ZnPc), joined by bulk heterojunction (BHJ) with dibenzotetrathiafulvalene (DBTTF). These structures are shown in Figure 1. Although over 70 metallic ions will fit into the Pc cavity, ZnPc has outstanding specific properties, including its high mobility and high environmental stability, as well as a suitable energy level and bandgap for biomedical optoelectronic applications [23,24,25]. Therefore, in this work, ZnPc was chosen, and the introduction of fluorine in the structure of the F_16_ZnPc was carried out to enhance their behavior as an *n*-type molecular semiconductor. In a similar manner as ZnPc, DBTTF is a practically plane molecule that tends to form segregated or uniforms piles of the mono- or two-dimensional type. The presence of DBTTF as a *p*-type semiconductor is due to its electron’s donor ability and to its almost coplanar structure with strong π-π overlapping and interactions S···S that facilitate the transport of charges. This allows an electronic transfer between molecules and propitiates the resultant properties in the ONs that are synthetized in the present study as anisotropic and associated with a preferential direction by which electric charges circulate. The ONs were obtained by a mixture based on green chemistry, using a reactor with controlled pressure and temperature. The Nylon-11 membranes were made by high-vacuum evaporation followed by thermal relaxation on different types of substrates, namely, Corning glass, monocrystalline silicon, and indium tin oxide (In_2_O_3_·(SnO_2_)x)-coated glass slides (glass-ITO). The chemical characterization of the membranes was carried out through infrared spectroscopy (IR), while the optical evaluation took place by ultraviolet-visible spectroscopy (UV-vis) and photoluminescence (PL). The characterization of the electric behavior was evaluated through current density-voltage (*J*-*V*) characteristics in the membranes, under different radiation conditions. Finally, the membranes were analyzed, after subjecting them to accelerated lighting conditions, in order to determine their stability with radiation and their possible application in molecular electronics. Introducing MPcs into Nylon-11 could give outstanding and effective properties in membrane applications [19,26,27,28,29,30,31]. Some examples of these applications include use as photosensitizers [32,33], for photocatalytic reactions [19,34], or as separation membranes by their permselectivity [35], although the information about the manufacture of semiconductor membranes that could be used in electronic devices as diodes (OLED) or organic transistors (OFET) is scarce. The technological importance of the present study lies in the proposal to manufacture membranes with a polymer matrix, constituted by ambipolar semiconductors of BHJ. A major disadvantage of molecular semiconductors is their low chemical stability; however, this can be improved using semiconductor membranes where the polymer matrix protects the semiconductor from the presence of environmental agents. Due to the great potential of MPcs in different applications, it is important to find alternatives that reduce their degradation.

## 2. Materials and Methods 

### 2.1. Fabrication of π-Conjugated Molecular Semiconductors 

Polyundecanolactam (Nylon-11: [-NH(CH_2_)_10_CO-]n), Zinc phthalocyanine (ZnPc: C_32_H_16_N_8_Zn), Zinc 1,2,3,4,8,9,10,11,15,16,17,18,22,23,24,25-hexadecafluoro-29H,31H-phthalocyanine (F_16_ZnPc: C_32_F_16_N_8_Zn) and dibenzotetrathiafulvalene (DBTTF: C_14_H_8_S_4_) were acquired from the commercial supplier (Sigma-Aldrich, Saint Louis, MO, USA). Due to their purity certificate, no additional purification was needed. The bulk heterojunction (BHJ) aims to achieve a continuous phase between semiconductors *p*-type and *n*-type that improve the efficiency of devices. The BHJ is formed by the mixture of these semiconductors in a common solvent, which generates dispersed phases and interconnects one to the other in a random distribution of the two semiconductors, among which there is a greater contact surface [36]. In the present study, ambipolar semiconductors are manufactured from the BHJ of *p*-type and *n*-type molecular semiconductors. The ambipolar molecular semiconductors were obtained by a mixture with a 1:1 mass ratio suitable for green chemistry, using a Monowave 50 reactor (Anton Paar GmbH, Graz, Austria) with controlled time, temperature and pressure. The reactor was operated using a borosilicate glass vial with an integrated pressure of 20 bar. Reactions were kept in the reactor for 35 minutes, and then slowly cooled in the reactor by lowering the pressure and temperature of the system. Subsequently, the filtering process was carried out, which included washing and vacuum drying. Decomposition points were obtained on a Melt-Temp II apparatus (Thermo Fisher Scientific Inc., Waltham, MA, USA). The particle semiconductors were chemically characterized by IR spectroscopy on a Nicolet iS5-FT spectrometer (Thermo Fisher Scientific Inc., Waltham, MA, USA) on a wavelength range of 4000 to 400 cm^−1^, using KBr pellets. 

Semiconductor molecular derivative from ZnPc **(ONs-1)**. 200 mg (0.35 mmol) of ZnPc was added to 200 mg (0.66 mmol) of DBTTF in 4 ml of absolute ethanol. Tdec. > 310 °C.

Semiconductor molecular derivative from F_16_ZnPc **(ONs-2)**. 200 mg (0.23 mmol) of F_16_ZnPc was added to 200 mg (0.66 mmol) of DBTTF in 4 ml of absolute methanol. Tdec. > 310 °C.

### 2.2. Membrane Fabrication and Characterization

Membranes were manufactured using the high-vacuum evaporation method, in three different substrates: (100) monocrystalline silicon (c-Si), Corning glass slides and indium tin oxide (In_2_O_3_·(SnO_2_)x) coated glass slides (glass-ITO) (Sigma-Aldrich, Saint Louis, MO, USA). To generate adhesion between the membrane and the substrate, silicon wafers were cleaned from the surface oxide layer with which they were covered, using a “p” solution (10 mL HF, 15 mL HNO_3_ and 300 mL H_2_O). The Corning glass and the glass with the conductor layer of the transparent oxide ITO were both washed in an ultrasonic bath, using chloroform, methanol, and acetone (Sigma-Aldrich, Saint Louis, MO, USA), consecutively. All the substrates were dried under a vacuum, before the deposition of the membranes into a vacuum chamber. The vacuum was accomplished through two pumps: a mechanical pump that generated an initial pressure of 10^−3^ torr and a turbo-molecular pump that generated the final deposition pressure of 10^−6^ torr. In addition to the two vacuum pumps, the equipment (INTERCOVAMEX, Morelos, México) used for the manufacture of membranes had two evaporation ports: the tantalum boat with Nylon-11 pellets inside was installed in the first port, and the tungsten boat with the semiconductor particles **ONs-1** and **ONs-2** was installed in the second port. The evaporation rate for the Nylon-11 was 23.5 Å/s and for the semiconductor particles was 2.7 Å/s. The pressure in the vacuum chamber was 1 × 10^−6^ torr throughout the manufacturing process. During the deposition processes, the thickness of the films was monitored with a high-resolution thickness monitor, with a quartz sensor (INTERCOVAMEX, Morelos, México). After deposition, in order to embed the semiconductor particles into the Nylon-11, the membranes were treated by annealing for thermal relaxation in air, at 140 °C, for 12 min. The membranes deposited over silicon were analyzed by IR spectroscopy and scanning electron microscopy (SEM). IR analysis of the infrared absorption spectrum was carried out with a Nicolet iS5-FT spectrometer (Thermo Fisher Scientific Inc., Waltham, MA, USA), and, for SEM, a Zeiss EVO MA 10 scanning electron microscope (Carl Zeiss AG, Oberkochen, Germany) was operated at 3-kV voltage. The dependence of the electric current to the voltage (*J*-*V*), using the four-point collinear probe method was evaluated using ITO-coated glass slides. A Keithley 4200-SCS-PK1 (Tektronix Inc., Beaverton, OR, USA) programmable voltage source, with auto-ranging pico-ammeter, and a Next Robotix (Comercializadora KMox, S.A. de C.V., Mexico City, México) sensing station were used for electrical characterization. The Keithley device was used in the voltage source mode and the current was measured. The distance between the electrodes of the four-point sensor used for the measurements was 1.59 mm. To evaluate the effect of the radiation over the degradation of membranes, an irradiation of 82V and 360 watts was generated with a Dukane 653 lamp (Topbulb: Semmer Lighting Company, Buffalo, NY, USA) for 4 h. After the accelerated radiation test, the *J*-*V* characterization in membranes was again analyzed. The evaluation of the optical behavior of the membranes deposited over Corning glass was performed by UV-vis spectroscopy with a Unicam spectrophotometer, model UV300 (Thermo Fisher Scientific Inc., Waltham, MA, USA), in the wavelength range of 200–1100 nm. Photoluminescence was measured over silicon in a Raman Micro spectrometer, Model: HR Evolution, (Horiba Scientific, Kyoto, Japan) and Argon Laser tuned at 488 nm. Both electrical and optical behavior and photoluminescence were compared to that of the thin films of molecular semiconductors **ONs-1** and **ONs-2**. Thin films were manufactured over the same type of substrates used in the manufacture of the membranes and using the same technique and high vacuum evaporation equipment. The films were structurally characterized by IR spectroscopy and SEM, and additionally, X-Ray diffraction was carried out using the θ–2θ technique in a Rigaku D-max 2100 diffractometer (Rigaku Americas Corporation, The Woodlands, TX, USA), using Cu Kα (λ = 1.5406 Å) at 30 kV, 20 mA. Silicon substrates were used for the characterization of the films by X-ray diffraction (XRD). In the thin films, the evaluation of electrical, optical, and photoluminescent behavior was similar to that performed on the membranes, namely, using the four-point collinear probe method, UV-vis spectroscopy, and photoluminescence, respectively.

## 3. Results and Discussion

The manufacture of membranes took place in different stages. The first stage was the characterization of ZnPc, F_16_ZnPc and DBTTF precursors (see Figure 1) in terms of their solubility, melting/decomposition temperatures, and IR spectroscopy. The second stage consisted of the fabrication and characterization of the semiconductor particles ONs. The third stage included the manufacture of membranes and their subsequent chemical characterization, optical properties evaluation, photoluminescence, and determination of electrical behavior. It is important to note that the optical and electrical behavior was compared to that of the semiconductors **ONs-1** and **ONs-2** without Nylon-11. This was performed to verify that the semiconductor properties had improved in the membrane. Finally, in the fourth stage, the study was conducted in accelerated lighting conditions, to determine the stability of the membranes against radiation and their possible application for electronic devices. 

To chemically characterize **ONs-1** and **ONs-2**, both in isolation and within the membranes, the IR spectroscopy was initially carried out in the KBr of the particles and in the chemical compounds from which they were synthesized. Table 1 illustrates the most representative signals for ZnPc and F_16_ZnPc, which is the band responsible for C-N stretching at 1283 ± 3 cm^−1^. In this table, the C=C benzene stretch is represented by the band 1607 cm^−1^_,_ while the band observed at 1333 cm^−1^ is attributed to C=N in the macrocyclic ring. The signals from 1163 ± 1 cm^−1^, 1084 ± 4 cm^−1^, and 1117 ± 1 cm^−1^ were assigned to in-plane C-H bending, while the band from 1483 ± 1 cm^−1^ is given by the C-H bending in aryl [37,38,39,40,41]. With regard to DBTTF, the signal is present at 1508 cm^−1^ for C-S, at 1151 cm^−1^ for C-H, and at 783 cm^−1^ for C=C [42]. Figure 1d presents the IR spectrum and Table 1 presents selected signals that correspond to the synthesized particles **ONs-1** and **ONs-2**: the referent to MPcs is present, the signal at 1284±3 cm^−1^ corresponds to the C-N stretching, the band at 1606 ± 3 cm^−1^ is attributed to the C=N bond in the macrocycle, and the signals corresponding to C-H at 1486 cm^−1^, 1162 ± 2 cm^−1^, 1083 ± 3 cm^−1^, and 1117 ± 2 cm^−1^. The presence of DBTTF, as an integral part of the particles, is proved by the appearance of bands at 1508, 1152, and 777 ± 1 cm^−1^, which are present in the spectra of **ONs-1** and **ONs-2**. Additionally, IR spectroscopy was used to identify the crystalline nature of ZnPc semiconductors, since the IR spectrum is dependent on the crystalline form [40,41]. Normally, phthalocyanines are arranged in crystalline forms, since aromatic rings are neatly stacked, so it was also reported that the α-form of MPc can be characterized by a band of around 720 cm^−1^, while β-form can be characterized by a band at a greater wave number, of around 778 cm^−1^ [40,41]. The difference between one type of crystalline structure and another is the angle formed between the symmetry axe and the stacking direction. The alpha crystals have a 26.5° angle, and the beta crystals have a 45.8° angle, with respect to the axis. According to the IR spectra for ZnPc and F_16_ZnPc (Figure 1d), phthalocyanine presents both crystalline forms and, after being mixed with DBTTF, both the α-form and the β-form remain for **ONs-1** and **ONs-2**.

**Figure 2:**
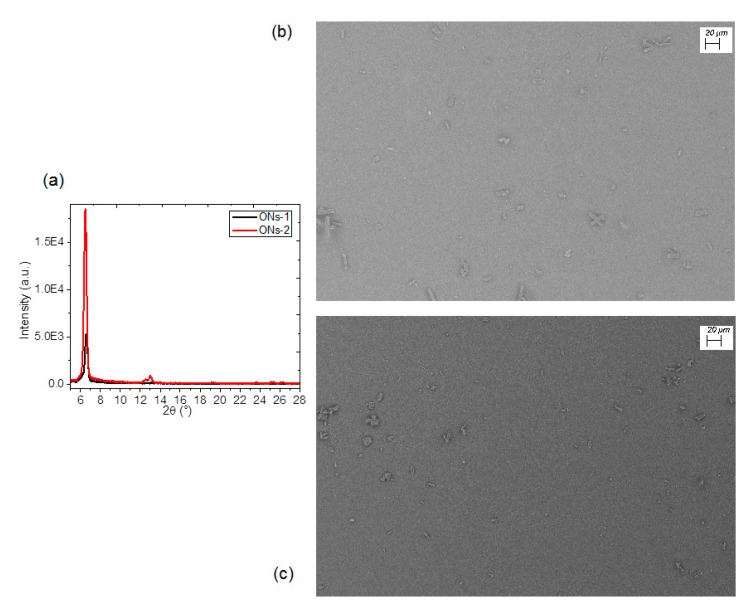
(**a**) X-ray diffraction patterns of the two thin films, and SEM images of (**b**) **ONs-1** and (**c**) **ONs-2** films at 500x magnification.

The organization type of the phthalocyanine and the DBTTF will determine the properties of the **ONs-1** and **ONs-2**. Therefore, in order to evaluate their optical and electric properties, they were deposited as thin films using the high-vacuum evaporation technique. The properties of the molecular semiconductors are better utilized for electronic uses in the form of thin films. The **ONs-1** and **ONs-2** films were analyzed by IR spectroscopy, in order to verify that, during the evaporation and posterior deposit, the material did not suffer chemical decomposition. The IR spectroscopy results indicated that **ONs-1** and **ONs-2** did not suffer decomposition. However, to determine the amorphous or crystalline nature of the films, X-ray diffraction was carried out. Figure 2a indicates the X-ray diffraction patterns of the two thin films deposited on silicon substrates, which makes the amorphous nature of these films evident, as there are probably small areas with α-form. The α-phase is characterized by the peak observed around 6.6°. This peak arises from the interlayer spacing of stacks of tilted molecules, which means that crystals are oriented with the MPc molecular plane perpendicular to the substrate [43,44]. The nature of the π bonds of the phthalocyanine, which are present in **ONs-1** and **ONs-2**, and the presence of fluorine in the F_16_ZnPc that constitutes **ONs-2** both promote the α-form. Although MPc and DBTTF are flat molecules, the X-ray diffraction analysis revealed the disorder and lack of molecular stacking within the particles, which is caused by variations in the interactions between phthalocyanine molecules and DBTTF. To complement the previous results, Scanning Electron Microscopy (SEM) was performed on the semiconductor films. According to the photomicrographs depicted in Figure 2b,c, both cases present a heterogeneous morphology that is constituted by some particles of irregular shape, deposited over a layer of the semiconductor. This morphology is a product of the high-vacuum evaporation method, which generates different thermal gradients along the deposit substrate. Thus, the semiconductor nucleates and grows heterogeneously. This technique was precisely selected for its ease in terms of generating amorphous and heterogeneous structures, which are characteristic of semiconductor membranes. 

**Figure 3:**
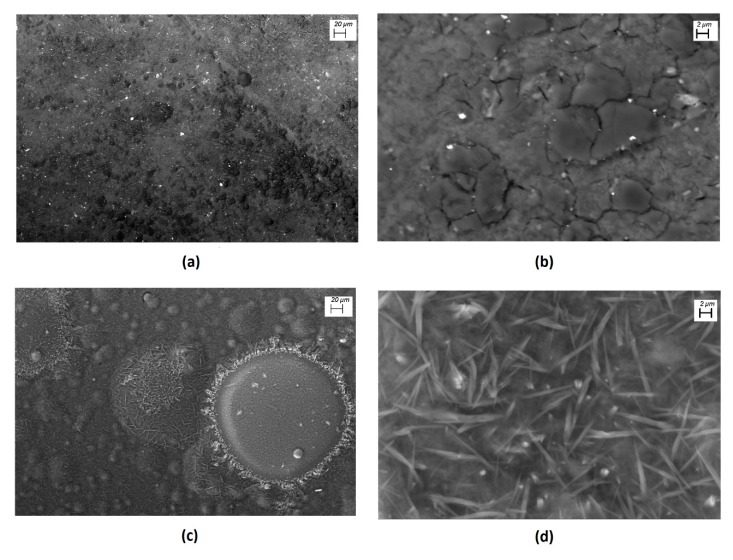
SEM images of (**a**) **membrane-1** at 500x, (**b**) **membrane-1** at 5000x, (**c**) **membrane-2** at 500x, and (**d**) **membrane-2** at 5000x magnification.

The membranes were manufactured by the high-vacuum evaporation method also conducted for the films. Subsequently, the thermal relaxation of Nylon-11 was carried out. After the manufacture of the membranes, IR spectroscopy was performed. The purpose of this was to verify the chemical stability of **ONs-1** and **ONs-2**, as well as the Nylon-11. High-vacuum evaporation is a method that requires the components of the membrane to pass by two high thermal gradients, the first of which allows the components of the membrane to change to the gas phase and the second thermal gradient allows another change to the solid phase, over the substrates. Table 1 presents the signals obtained during the characterization of the membranes by IR spectroscopy. All the signals that corresponding to the particles were observed, and the signals of Nylon-11 were also observed. From this last observation, the bands at 3306 and 3083 ± 2 cm^−1^ correspond to the stretching vibrations for N-H, and the band around 1654 ± 3 cm^−1^ is assigned to the C=O vibrations in the polymeric matrix [9]. The results obtained suggest that, during the manufacture of the membranes, there was no degradation of their components and no signals corresponding to impurities were generated during the fabrication of the membranes, which indicates that the high-vacuum evaporation method generates a high-purity membrane. The morphology of the membranes was studied by means of SEM, and Figure 3 shows the surface images at different magnifications. The micrographs obtained revealed the roughness of the membranes’ skin layer, and the way in which particles are embedded in the Nylon-11 matrix was observed in both membranes. The membrane is formed by particles, most of which are rounded structures of different sizes. This difference in size is because, during deposition of the membrane, heterogeneous nucleation occurs, and growth preferential directions are generated in the nuclei of ONs. As a result, each particle grows at its own speed. Additionally, due to the lower free energy per unit volume of the sphere, the nuclei of the membrane deposited on the substrate resembles a geometry (see Figure 3c,d) that, when it grows, is maintained as the deposit advances and the membrane is formed. The growth and nucleation process generated by ONs particles depends directly on the structure of the molecular semiconductor (**ONs-1** and **ONs-2**) and the thermic gradient between the substrate and the system ONs/Nylon-11 [9]. Thermal relaxation is also worthy of discussion, as during heating Nylon-11, fibers undergo an elongation that allows ONs to penetrate the matrix of the membrane. As the polymer gets cold, the fibers contract and the ONs remain surrounded by contracted fibers. It is noteworthy that the polymeric matrix is evenly distributed over the substrate, as this distribution prevents the dissipation of the electromagnetic radiation that may affect optoelectronic behavior in membranes. This must be proved with the electrical analysis in the membranes **ONs-1**/Nylon-11 (**membrane-1**) and **ONs-2**/Nylon-11 (**membrane-2**).

**Figure 4:**
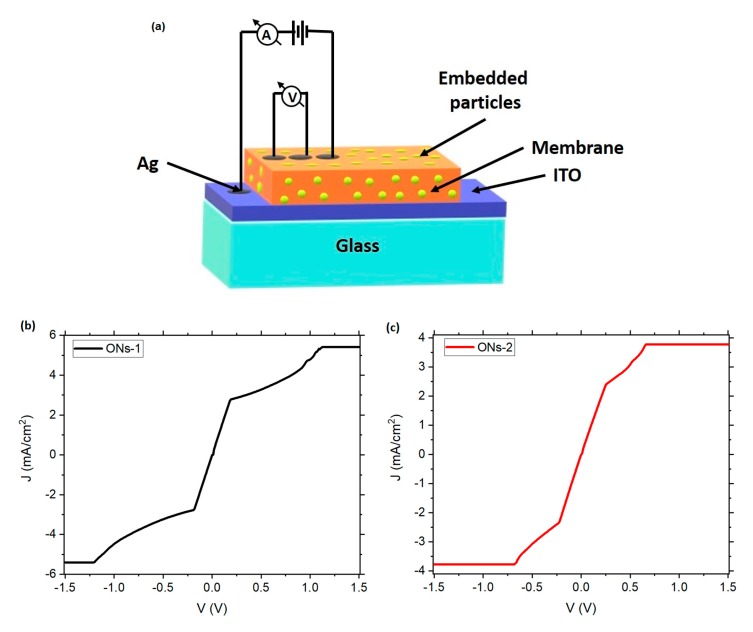
(**a**) Schematic structure of the electrical measurements on membranes and *J-V* characteristic of (**b**) **ONs-1** and (**c**) **ONs-2** films.

Evaluation of the electrical behavior was performed in the films and in the membranes, in order to assess the effect of Nylon-11 on the electrical properties of the **ONs-1** and **ONs-2** semiconductors. Initially, the electrical behavior of the ONs was determined by a current density-voltage (*J*-*V*) characterization, in systems integrated by the **ONs-1** and **ONs-2** films, on a glass substrate coated with the transparent conductor ITO (In_2_O_3_·(SnO_2_)x). The measurements were carried out using the four-point collinear probe method, with ITO as anode and a silver electrode as cathode. The same structure was used with the membranes (see Figure 4a). *J-V* characteristics of both ONs films are very similar, and their behavior can be explained by charge trapping. Due to their amorphous nature, these films present a high density of trap states that change their electronic behavior. These traps (deep localized states) can capture injected carriers that would otherwise contribute to conduction; therefore, an SCLC (space-charge-limited conduction) regime arises. Hence, an ohmic behavior is observed at low voltages. However, when the voltage increases, a charge accumulation is generated in the ONs films and the current is dominated by charge carriers that are injected from the contacts. If the voltage is further increased, a quadratic behavior can be observed, and finally, in the limiting case, when there is an anomalous dispersion or defected states, the current may saturate, as in this case [45,46] (see Figure 4b,c).The main difference in the electrical behavior is that **ONs-2** film has a saturation at a lower voltage than the **ONs-1** film. Another point to notice is the high current density values obtained in comparison to other results, e.g., [47,48]. The high DBTTF doping may indeed be related to the high current density values, as the dopant´s presence is correlated with a substantial increase in conductivity [49,50]. During the ohmic regime, the transport of charges along the films is caused by the presence of the phthalocyanines ZnPc and ZnPcF_16_ rich in π electrons. These macrocycles are constituted by energy-accessible orbitals for the transport of charges. Once the charge is at the conjugate molecule, it is quickly delocalized on the π system. This process results in conduction, since electronic delocalization facilitates the transport of charges between molecules, due to the greater spatial overlap of the delocalized charge with the electronic states of adjacent DBTTF molecules. 

**Figure 5:**
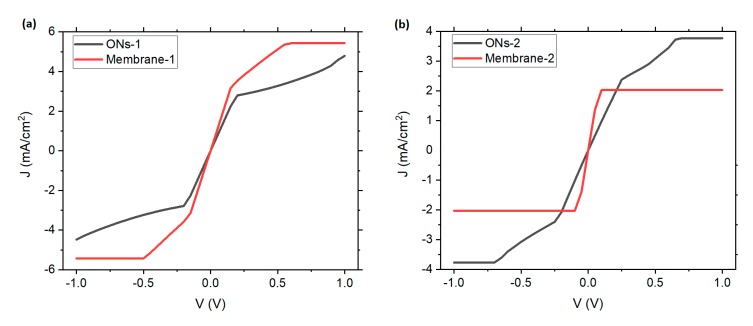
*J*-*V* characteristic of (**a**) **ONs-1** film and **membrane-1** and (**b**) **ONs-2** film and **membrane-2**.

It is extremely important to preserve the electric behavior of ONs after coating them with Nylon-11. The comparison of the *J*-*V* characteristics of the ONs films and the ONs membranes are presented in Figure 5. In the case of **membrane-1**, not only did it maintain its electrical characteristics, the ohmic conduction was also slightly improved. This also occurred for **membrane-2**; however, the saturation of the membrane was more abrupt than in the film. Thus, it is possible that, when the **ONs-2** is coated, the molecules adopt a structure in which more charges are trapped. The SCLC model was used to verify the charge carrier mobility of the membranes [23,51,52,53,54,55]:

(1) $$\;J=\frac{9}{8}\frac{\mu \varepsilon V^{2}}{d^{3}},$$

where *μ* is the hole mobility, *J* is the current density, *V* is the applied voltage, and *d* is the film thickness, $\varepsilon =\varepsilon_{0}\varepsilon_{r}$ where $\varepsilon_{0}$ is the permittivity of free space and $\varepsilon_{r}$ is the relative permittivity of the material, in this case, $\varepsilon_{0}=8.85\times 10^{-12}\frac{F}{m}$ and $\varepsilon_{r}=5$ for phthalocyanine derivatives [23,51,52,53,54,55]. The thickness of **membrane-1** is 74.5 nm and the thickness of **membrane-2** is 126 nm. The calculated mobilities of **membrane-1** and **membrane-2** are $4.91\times 10^{-6}\frac{cm^{2}}{Vs}$ and $1.92\times 10^{-5}\frac{cm^{2}}{Vs}$, respectively. These *μ* values are in the range reported for ZnPcs (10^−6^ cm^2^V^−1^s^−1^) [23]. The effect of DBTTF as a *p*-type dopant is significant, as it promotes the transport of charge between its stacks that are segregated with phthalocyanine. In addition, their sulfur atoms help the valence electrons of the molecule to be located above and below the middle plane of the molecule in delocalized π orbitals, promoting conductivity. Another important aspect to consider is the symmetry of the *J*-*V* curves, which is maintained in the membranes and which is related to the ambipolarity of the **ONs-1** and **ONs-2** semiconductors. In films and membranes, the ITO and the silver can be exchanged as an anode and cathode, respectively, without affecting the transport of charges.

**Figure 6:**
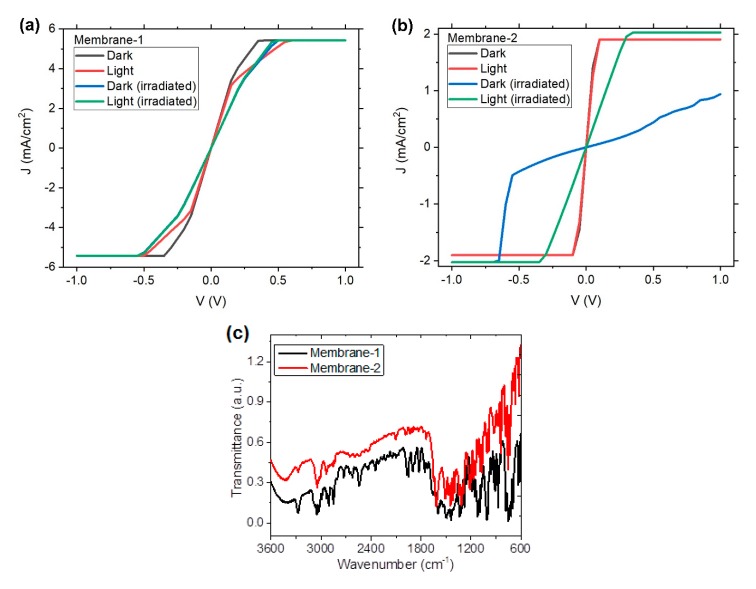
*J*-*V* characteristic of (**a**) **membrane-1** and (**b**) **membrane-2**, compared in natural light and darkness conditions. (**c**) IR spectrum for membrane after irradiation.

To evaluate the photoelectric properties of the membranes, the *J-V* characteristics were obtained in both natural light and darkness conditions. Furthermore, the membranes were subjected to a degradation test with a 360-Watt incandescent lamp for 4 h. Figure 6 presents a comparison of the membranes in different light conditions, before and after a degradation test. Figure 6a illustrates that the behavior of **membrane-1** is very similar in both light and darkness, even when the membrane is under irradiation. The same occurs in the case of **membrane-2** before degradation, and the behavior is practically the same in both lighting conditions. Therefore, the illumination conditions do not generate electrical excitations and, in turn, their use in photovoltaic applications could require further modification of the membranes. However, there was an alteration in the behavior of **membrane-2** after irradiation (see Figure 6b). First, the irradiated membrane did not have the same behavior in darkness and light conditions. With natural light the *J*-*V* curve was very similar to the membrane without treatment, but with a slight decrease of conductivity in the ohmic region. In darkness, however, the decrease in conductivity was considerable, even if the ohmic behavior was present over a wider voltage range. As such, its electrical properties were affected. It has been suggested that the contrast, compared with **membrane-1** after irradiation, is a result of fluorine substitution in ZnPcF_16_ of **membrane-2**. Apparently, this has a great impact on molecular electron affinity in the semiconductor, as well as on the molecular thermal motion that is induced by the irradiation at the surface [56]. Therefore, the irradiation does not affect the polymer chains of Nylon-11 that are linked by covalent bonds, but it does affect the phthalocyanine fluoride and the stacking between this and DBTTF. The result is that the charges do not have enough energy to move between the phthalocyanine and DBTTF molecules. When the membrane is illuminated, a photoexcitation is carried out that increases the conductivity. To analyze the possible degradation of Nylon-11 and the molecular semiconductor particles, the presence of the main functional groups was evaluated by IR spectroscopy. Figure 6c illustrates the IR spectrum after irradiation. The curves present the signs that refer to the stretching vibrations for N-H (around 3306 and 3083 cm^−1^) and the band assigned to the C=O vibrations (around 1636 cm^−1^) in the Nylon-11. Based on IR spectroscopy, it is possible to conclude that there was no chemical decomposition of the nylon in either the membrane or in the semiconductor particles. This is due to the presence in the spectra of Figure 6c of the bands corresponding to C-C, C=N and C-N vibrations in the phthalocyanines and the signals referent to the DBTTF around 1508, 1152, and 777 cm^−1^. On the other hand, in the region between 3300 and 3600 cm^−1^, the presence of the characteristic signals of the hydrogen bridge of water, which can affect the semiconductor properties of the membranes, is observed. For the above, it is necessary to obtain the optical bandgap (E_g_), which is a parameter that allows to establish the capacity of the semiconductor to transport electric charges.

**Figure 7:**
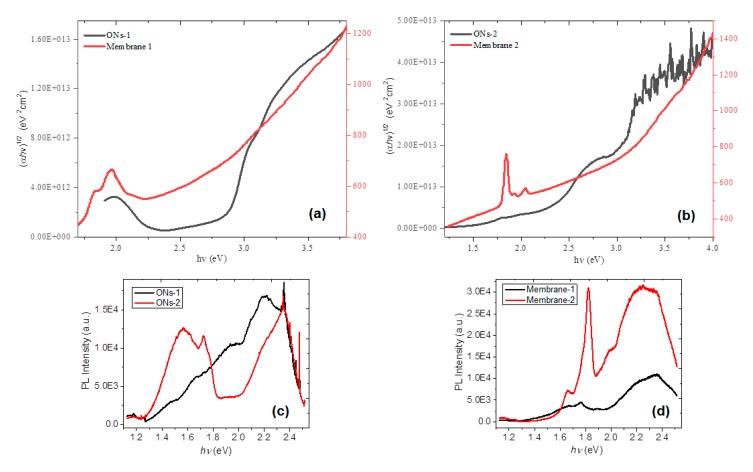
Plot of (αhν)1/2 vs. photon energy of the (**a**) **ONs-1** film and **membrane-1**, (**b**) **ONs-2** film and **membrane-2,** and Photoluminescence spectra of (**c**) films and (**d**) membranes.

The E_g_ controls the efficiency of the light absorption in the membranes. The E_g_ associated with the semiconductor membranes is determined through an extrapolation of the linear trend, which is observed in the spectral dependence of (αhν)^1/2^ over a limited range of photon energies (hν) [57]. The absorption coefficient (α) and the frequency (ν) are experimentally obtained from the UV-vis spectrum and the thickness of the film, while h is Planck’s constant. The coefficient α near the band edge shows an exponential dependence upon photon energy, which usually obeys the Urbach relation $\alpha h\nu =\beta {\left(h\nu -E_{g\;}\right)}^{n}$, and ½ is a number characterizing the transition process in amorphous semiconductors [57]. Figure 7a,b visualizes the dependence of (αhν)^1/2^ over photon energies (hν) for the ONs films and the membranes. The E_g_ for organic semiconductors is generally found between 1.5 and 4.0 eV [58], so the ONs could be deposited as membranes, since their E_g_ is within this range: 2.8 eV for **ONs-1** and 2.3 eV for **ONs-2**. Conversely, the organic **membrane-1** has 2.3 eV and **membrane-2** has 2.4 eV, which is a clear indication that Nylon-11 does not affect the semiconductor properties of the particles **ONs-2**. When the semiconductor particles of **ONs-2** are introduced in Nylon-11, no effect was observed in the molecular semiconductor. Indeed, the E_g_ remained practically the same. In the case of particles **ONs-1**, the results change, since the E_g_ decreases in 0.5 eV when these particles are introduced into Nylon-11. M**embrane-1** presents a lower E_g_ than that presented by **ONs-1**; this indicates the effectiveness of Nylon-11 as the matrix of the membrane. The excellent mechanical properties of Nylon-11 probably promote the compression at a molecular level between the dispersed hetero-junction ZnPc-DBTTF, which does not occur in the hetero-junction F_16_ZnPc-DBTTF due to the presence of fluorine atoms. A suitable and continuous compatibility of molecular semiconductor **ONs-1** at the polymer is present, which generates a higher organization in the BHJ. Additionally, in the graphs of the membranes in Figure 7a,b, two peaks are visible at 1.8 and 2.0 eV. The first of these refers to the presence of ZnPc, and the second refers to DBTTF, which is apparently absorbed at low energies that correspond to red and orange, respectively. These lower energy peaks can result in an intramolecular charge transfer characteristic along the semiconductor [59], which represents a charge transfer transition from the donor DBTTF to π orbitals that are associated with the acceptor phthalocyanine or even with the same ion Zn. 

The membranes were also analyzed by photoluminescence (PL) at room temperature, to complement their optical study. These results were compared to those obtained for films **ONs-1** and **ONs-2** (Figure 7c,d). For the film with **ONs-1**, the bandwidth in Figure 7c indicates the presence of several very similar energy levels, which converge in that single band. In the case of the film with **ONs-2**, two high-intensity bands were observed, whose separation suggests that they are generated by different compounds (F_16_ZnPc and DBTTF). The band between 1.3 and 1.8 eV is constituted by two peaks, which indicates at least two energy levels, while the inflection in the highest peak suggests a third level. Comparing the PL graphs of membranes to that of the films reveals that in the case of **membrane-1**, the PL is practically the same, although with lower intensity. **Membrane-2** presents an intense PL in the visible range of spectrum, which is practically double that which is presented by the thin film of **ONs-2**. These results are indications that the total PL intensity increases when the F_16_ZnPc is present (**membrane-2**) and decreases for ZnPc (**membrane-1**), which suggests that F_16_ZnPc generates higher PL intensity. This greater intensity is present with lower energy transitions, probably of the n-π type. The variation of the PL signal as a function of position on the sample was minimal, which was consistent with the uniformity found for the thickness of the films on the entire substrate [60]. On the other hand, Figure 7d reveals one peak at 1.66 eV (~751 nm), which is characteristic of Nylon-11, and which disappears for **ONs-1** and **ONs-2** films in Figure 7c. The peak of around 1.8 eV (~680 nm), which is associated with the π*-π relaxation transitions of the molecular semiconductor, is red-shifted for the membrane. This is related to the π-π* stacking of the conjugated Pc as the semiconductor particles are introduced in the Nylon-11, which infers molecule aggregation [9]. 

## 4. Conclusions

Using high-vacuum evaporation followed by thermal relaxation, semiconductor membranes were manufactured consisting of ZnPc-DBTTF and F_16_ZnPc-DBTTF particles dispersed in Nylon 11. The presence of the main bonds in the π-conjugated molecular semiconductors and in the Nylon-11 were verified by IR spectroscopy. This is a sign of the chemical stability of the membranes. These membranes combine polymer properties with organic semiconductor properties, and according to the IR spectroscopy, they also provide a barrier effect for molecules of environmental agents. A comparison of the *J*-*V* characteristics of the ONs films and the ONs membranes reveals that **membrane-1** not only preserved the electrical characteristics but also slightly improved the ohmic conduction. The same occurred for **membrane-2**; however, the saturation of the membrane was more abrupt than in the film. The calculated mobilities of **membrane-1** and **membrane-2** were $4.91\times 10^{-6}\frac{cm^{2}}{Vs}$ and $1.92\times 10^{-5}\frac{cm^{2}}{Vs}$, respectively, which is within the range reported for ZnPcs. Furthermore, after fatigue conditions, the electrical behavior of the proposed semiconductor membranes was preserved. The E_g_ for semiconductor **membrane-1** is 2.3 eV and for **membrane-2** is 2.4 eV, which indicates that Nylon-11 can promote the semiconductor properties of the semiconductor particles and can be used in the manufacture of semiconductor membranes.

## Figures and Tables

**Table 1:** Characteristic FT-IR signals (cm^−1^).

Sample	ν(C-N)	ν(C=C)	ν(C=N)	ν(C-H)	α/β- form	ν(C-S), ν(C-H), ν(C=C)	Nylon 11 ν(N-H)	Nylon 11 ν(C=O)
ZnPc (KBr)	1286	1607	1332	1484, 1164, 1118, 1088	778, 724	-	-	-
F_16_ZnPc (KBr)	1280	1607	1332	1483, 1162, 1117, 1080	770, 720	-	-	-
DBTTF (KBr)	-	-	-	-	-	1508, 1151, 783	-	-
ONs-1 (KBr)	1282	1603	1332	1486, 1160, 1119, 1080	776, 718	1508, 1152, 778	**-**	**-**
ONs-1 membrane	1283	-	1336	1484, 1166, 1119, 1080	-	1156	3306, 3085	1656
ONs-2 (KBr)	1287	1609	1331	1486, 1164, 1115, 1085	773, 723	1508, 1152, 777	-	-
ONs-2 membrane	1287	-	1336	1484, 1165, 1118, 1080	-	1504, 1150, 778	3306, 3083	1656
